# Nanocellulose-Block Copolymer Films for the Removal of Emerging Organic Contaminants from Aqueous Solutions

**DOI:** 10.3390/ma12020230

**Published:** 2019-01-11

**Authors:** Jairo Herrera-Morales, Taylor A. Turley, Miguel Betancourt-Ponce, Eduardo Nicolau

**Affiliations:** 1 Department of Chemistry, University of Puerto Rico, Río Piedras Campus, 17 Ave. Universidad STE 1701, San Juan, PR 00925-2537, USA; jairo.herrera@upr.edu (J.H.-M.); miguel.betancourt3@upr.edu (M.B.-P.); 2 Molecular Sciences Research Center, University of Puerto Rico, 1390 Ponce De Leon Avenue, Suite 2, San Juan, PR 00931-3346, USA; taylor.a.turley@students.jsums.edu; 3 Department of Chemistry, Jackson State University, 1325 J. R. Lynch St. P.O. Box 17910, Jackson, MS 39217-0510, USA

**Keywords:** cellulose nanofibers (CNFs), poly(4-vinylpyridine-b-ethylene oxide) (P4VP-PEO), emerging organic contaminants (EOCs), sulfamethoxazole (SMX)

## Abstract

The prevalence of emerging organic contaminants (EOCs) in ground and surface water has sparked the search for more effective methods to remove EOCs from the environment. In pursuit of a solution for this environmental concern, herein we present the development of reusable films based on cellulose nanofibers (CNFs) and the block copolymer, poly(4-vinylpyridine-b-ethylene oxide) (P4VP-PEO) to adsorb sulfamethoxazole (SMX) as an EOC model compound. We hypothesize that the adsorption of SMX was achieved mainly by π-π interactions between the pyridine functionalities of the block copolymer and the electron deficient phenyl group of the SMX. Preceding preparation of the films, CNFs were modified with the alkoxysilane trimethoxy(2-phenylethyl)silane (TMPES) to increase their stability in aqueous solution. After the addition of P4VP-PEO, the process was completed by filtration followed by oven-drying. XPS and FTIR were employed to confirm the addition of TMPES and P4VP-PEO, respectively. Adsorption batch experiments were performed in aqueous solutions of SMX at a neutral pH, obtaining adsorptions of up to 0.014 mmol/g in a moderate time of 60 min. For the reusability tests, films were immersed in ethanol 95 wt.% to elude the adsorbed SMX, rinsed with deionized (DI) water, and dried at room temperature to be reused in a new adsorption cycle. We found that this new composite material could be reused several times with negligible loss of adsorption capacity. The films presented have been shown to be of substantial importance for water remediation as they find direct application in the adsorption of electron deficient aromatic compounds and are reusable.

## 1. Introduction

Emerging organic contaminants (EOCs) are frequently present in aquatic ecosystems [1]. EOCs consist of compounds that originate from sources such as: pharmaceuticals, food additives, industrial compounds, personal care products, by-products from water treatment [2], and wastewater from hospital effluent and/or chemical manufacturing plants, as well as livestock, and agriculture [3]. Typically, wastewater treatment plants employ the processes of flocculation-filtration and oxidation-chlorination to reclaim water. However, flocculation-filtration methods are not able to completely remove EOC’s due to ineffective physical interaction. Moreover, chlorination has been found to produce residual by-products that could be potentially be more toxic to the environment than the parent compound [2,4]. Different approaches have been proposed to deal with the remediation of EOCs, particularly the use and modification of novel carbon-based materials such as graphene [5] and carbon nanotubes (CNTs) [6]. Nevertheless, the ecotoxicity of such materials could make them undesirable for water remediation [7,8]. Another carbon-based material that is of interest for application in water remediation is nanocellulose (NC). NC exists in different forms such as, cellulose nanocrystal (CNC), cellulose nanofibers (CNF), and bacterial cellulose (BC) [9]. These materials are environmentally benign, stable, and have a high aspect ratio and high surface area-to-volume ratio [9,10]. NC is typically used as a supporting material due to its unique structure consisting of a long chain of glucose with an outskirt of hydroxyl groups [11]. These hydroxyl groups can be chemically modified in order to promote selective interactions with a wide variety of contaminants. NC has been extensively used for the adsorption of heavy metals [12,13,14,15] and dyes [16,17,18] by means of electrostatic interactions between the aqueous ions and charged functional groups added to the NC surface. Unfortunately, this approach is not suitable for the adsorption of organic contaminants of low polarity. This drawback has been addressed by the preparation and modification of highly porous NC aerogels [19] that are effective in the removal of hydrophobic contaminants by entrapment [20]. However, preparation of such materials requires the use of expensive and time-consuming supercritical freeze-drying or solvent exchange methods. Moreover, the remediation mechanism involves diffusion into the structure which could make the regeneration and reutilization of the material difficult. A feasible strategy to enhance the adsorption of NC against organic contaminants involves functionalization with materials that address the adsorption via more specific interactions. This improvement can be accomplished by the addition of block copolymers (BCPs). BCPs are a class of polymer that has gained special attention in recent years for remediation [21,22,23]. Block copolymers are potential candidates for modification of NC intended for water remediation due to their enhanced functional properties. This is possible because these polymers can be configured into a nearly infinite number of molecular architectures based on the composition and molecular weights of their constituent monomers [24,25]. This versatility can allow the BCPs to interact with organic contaminants through physical and chemical interactions, such as electron donor-acceptor, that can be reversed allowing reuse of the material [26].

The handling of a material after adsorption is an important factor that determines its feasibility for large-scale operation. It is well known that NC is easily dispersed in aqueous solutions due to its hydrophilicity [27], and so requires high-speed centrifugation to remove it from solution. Nevertheless, the addition of hydrophobic moieties to the NC surface has proved to be effective in overcoming this shortcoming. There are several reports of alkoxysilanes [28] being used to stabilize NC in water, and at the same time imparting super hydrophobic characteristics for the removal of oil from water [29,30,31].

Here, we present a novel material prepared by the modification of CNFs with the alkoxysilane trimethoxy(2-phenylethyl)silane (TMPES) and the addition of poly(4-vinylpyridine-b-ethylene oxide) (P4VP-PEO). This material was used to fabricate films by a simple vacuum-filtration method and then employed for the adsorption of sulfamethoxazole (SMX) as a model EOC in water. CNFs are composed of fibers with diameters in the range of 5–50 nm and lengths of several micrometers that can form intricate structures due to crosslinking which ultimately define the films [32]. These films are completely stable in water since the phenyl groups provided by the silane confers hydrophobicity to the surface of the CNFs. Additionally, the P4VP-PEO present in the structure can interact with SMX by means of electron-donor-acceptor (EDA) interactions to complete the adsorption process. Finally, these films are reusable after immersion of the material in ethanol to elude the SMX.

## 2. Experimental Procedures

### 2.1. Materials

TMPES 98%, glacial acetic acid, and SMX of an analytical standard were purchased from Sigma-Aldrich (St. Louis, MO, USA). CNFs (3 wt.% aqueous slurry) were acquired from the University of Maine Process Development Center (Orono, ME, USA). P4VP-PEO (20–5 kD) was purchased from the Polymer Source Inc. (Dorval, QC, Canada). All reagents were used without further purification.

### 2.2. Preparation of Composite Films

#### 2.2.1. Modification of CNFs with TMPES and P4VP-PEO

TMPES was hydrolyzed in a polypropylene container by dissolving TMPES (2:1 with respect to the mass of CNFs) under magnetic stirring in ethanol/water 80:20, and by adding a small drop of acetic acid. Hydrolysis was completed after 2 h at room temperature. During the hydrolysis of TMPES, the CNFs solution was dispersed in ethanol/water 80:20 with the addition of a small drop of acetic acid in order to match the conditions of the hydrolysis of TMPES. Once hydrolysis of the alkoxysilane was completed, the CNFs solution was added to the TMPES solution under continuous stirring, and was then left to react for 24 h. CNFs solutions containing P4VP-PEO were prepared following a similar procedure as preparation of the TMPES-modified CNF. In this case, P4VP-PEO solid was dissolved in CNFs solution at a ratio of 1:1, then added to the previously hydrolyzed TMPES under continuous stirring to obtain a final ratio of 2:1:1 TMPES:CNFs:P4VP-PEO. The reaction was completed after 24 h of stirring at room temperature.

#### 2.2.2. Preparation of TMPES-Modified CNFs Films and TMPES and P4VP-PEO-Modified CNFs Films

Once the solution was completely synthesized, ultrasound was employed to obtain a homogeneous dispersed mixture after 10 min. Using vacuum filtration, it was possible to separate the solid materials and obtain the films. To successfully obtain the films without breakage, a nylon filter (0.47 µm) was first placed into the vacuum filtration system, and then the solution was added. To avoid shrinkage after filtration, each film was dried with nitrogen gas while inside the vacuum filtration system. After this, the film was peeled off the nylon filter, placed in an oven at 110 °C for 2 h, and then stored in petri dishes (sealed with paraffin sealing film) for further use.

#### 2.2.3. Preparation of CNFs Films without Modifications

CNFs films without modifications were prepared by dispersing CNFs in water to obtain 1 wt.% suspension. A small drop of acetic acid was added to the dispersion in order to attain the same acidic conditions of the CNFs modification. Suspension was stirred for 24 h and homogenized with ultrasound sonication for 10 min. Preparation of the actual films was performed following the same procedure for those modified with TMPES and P4VP-PEO. For that, vacuum filtration was used to obtain the films over a 0.47 µm nylon filter, peeled off, and dried in an oven at 110 °C for 2 h.

### 2.3. Characterization of Modified CNFs Films

#### 2.3.1. Fourier-Transform Infrared (FTIR) Spectroscopy

Modified CNFs films were characterized by Fourier-transform infrared (FTIR) spectroscopy using a Bruker Tensor 27 attenuated total reflectance (ATR) spectrometer (Billerica, MA, USA). The spectral width ranged from 400–4000 cm^−1^ for 32 accumulation scans and 4 cm^−1^ of resolution.

#### 2.3.2. X-ray Photoelectron Spectroscopy (XPS)

X-ray photoelectron spectroscopy (XPS) binding energy was obtained using a PHI 5600 spectrometer equipped (Physical Electronics, Chanhassen, MN, USA) with an Al Kα mono and polychromatic X-ray source operating at 15 kV, 350 W with a pass energy of 58.70 eV.

#### 2.3.3. Scanning Electron Microscopy

In order to assay the surface morphology of the films, we used a JEOL 6480LV scanning electron microscope (SEM, JEOL, Tokyo, Japan) in the secondary electron imaging (SEI) mode and 15 kV accelerating voltage.

#### 2.3.4. Contact Angle Measurements

The wettability of the films was tested by contact angle measurements performed with the Krüss drop shape analyzer DSA25S (Krüss Optronic, Hamburg, Germany) at room temperature. A 1 cm^2^ piece of CNFs, TMPES-modified CNFs, or TMPES and P4VP-PEO-modified CNFs film was fixed to the stage of the instrument using carbon tape. To start the analysis, a 4.50 µL DI water droplet was released from a syringe with a 25-gauge flat needle (0.51 mm inner diameter, 0.26 mm outer diameter) onto the surface of the sample. Images of the drop were recorded every 0.5 s up to 120 s (to avoid changes due to evaporation of the drop) and analyzed in real-time using Advance software (version 1.8).

### 2.4. Adsorption Batch Experiments of SMX using TMPES-Modified CNFs Films and TMPES and P4VP-PEO-Modified CNFs Films

#### 2.4.1. Adsorption as a Function of Time

The adsorption capacity of the films, with or without P4VP-PEO, was measured after placing them in contact with a SMX solution for 1, 10, 30, 60, and 240 min, respectively. 10 mL of 25 ppm SMX solution was used for each sample and shaken at 250 rpm for the stipulated equilibration time. The absorbance of each sample solution was collected before and after the adsorption using a Thermo Scientific Genesys 10S UV-Vis spectrometer (Waltham, MA, USA). Using the absorbance values, it was possible to calculate the SMX concentrations and then the equilibrium adsorption amount of the films using the following equation:

(1) $$q_{e}=\frac{\left(C_{0}-C_{e}\right)V}{w}$$

where *q*_e_ is the equilibrium adsorption amount (mg/g), *C*_0_ is the initial concentration of SMX (mg/L), *C*_e_ is the equilibrium concentration of SMX after the adsorption (mg/L), *V* is the volume of the SMX solution, and *w* is the mass of the modified CNFs. Experiments were performed in triplicate at 25 °C.

#### 2.4.2. Adsorption Isotherm Experiments

Batch adsorption isotherm tests were carried out in triplicate using TMPES-modified CNFs films with and without P4VP-PEO. The prepared solutions ranged from 1–100 ppm, and the contact time was set to 1 h at neutral pH and room temperature. 10.0 mL of each SMX solution was transferred to 50 mL Falcon tubes and a piece of the film was added to the solution. Then, the samples were placed in a shaker at 250 rpm and 25 °C. The absorbance of each sample solution was collected before and after the adsorption and measured using UV-Vis. The equilibrium adsorption amount, *q*_e_ of the films was calculated using the equation mentioned in Section 2.4.1. The resulting *q*_e_ was plotted as a function of the SMX equilibrium concentration (*C*_e_) to perform a fitting with the Freundlich mathematical model.

#### 2.4.3. Reusability Testing

In order to assay the reusability of the TMPES and P4VP-PEO-modified CNFs films, samples were immersed in 95 wt.% ethanol to elude the adsorbed SMX. This process was performed at least 5 times in triplicate for samples used in 1 h batch adsorption experiments with 10 mL of 25 ppm solution of SMX. The same elution procedure was also applied to control samples. The elution time in ethanol was set at 1h in continuous shaking of 250 rpm and 25 °C. Then, films were rinsed with 200 mL of water to remove the remnant ethanol, dried with compressed air until constant weight and used for another batch adsorption cycle. The equilibrium adsorption amount was calculated and plotted as a function of the cycles.

## 3. Results and Discussion

### 3.1. Modification of CNFs with TMPES and P4VP-PEO

**Figure 1:**
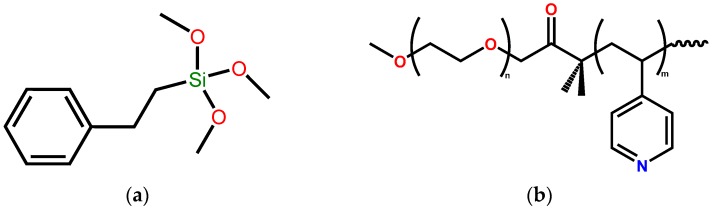
Molecular structure of TMPES (**a**) and P4VP-PEO (**b**).

Although alkoxysilanes with a wide range of functionalities have been continuously used as coupling agents to promote the adhesion of different polymeric surfaces [28], in this investigation, we aimed to enhance the stability of CNFs in aqueous solution. Previously, we prepared composites of nanocellulose grafted with a block copolymer intended for the adsorption of EOCs. This composite was easily dispersed in aqueous solution, making removal after the adsorption difficult and time-consuming [33]. Modifying the surface of CNFs with a hydrophobic functionality limits the solvation of the cellulose making it stable against dispersion. A silane compound, TMPES, was selected (Figure 1a) mainly due to its hydrophobic phenyl arm that is separated from the silane by two carbons, which is likely to help in lowering the steric repulsion during the reaction.

Due to the lower acidity of cellulosic hydroxyl groups present in CNFs, TMPES would have been unable to form covalent bonds on the surface of the CNFs [34]. For that reason, it was necessary to first hydrolyze the trimethoxy groups in TMPES to form silanols. This step also promotes the formation of –Si–O–Si– bonds that are resistant to hydrolysis, and are consequently unable to react with CNFs. Brochier-Salon et al., studied the kinetics of the hydrolysis of several alkoxysilanes (TMPES included) under acidic conditions using ^29^Si NMR. This study found that after approximately 3 h ca. 92% of silanol sites were still available with some formation of –Si–O–Si– groups [35]. Based on these findings, we carried out the hydrolysis for 2 h to avoid the presence of –Si–O–Si–. Thus, the resulting silanols would be available to interact with the hydroxyl groups on the surface of the CNFs via hydrogen bonds. These interactions are reversible, but heat treatment at 110 °C for 2 h is sufficient to form the –Si–O–C– bonds [28].

**Figure 2:**
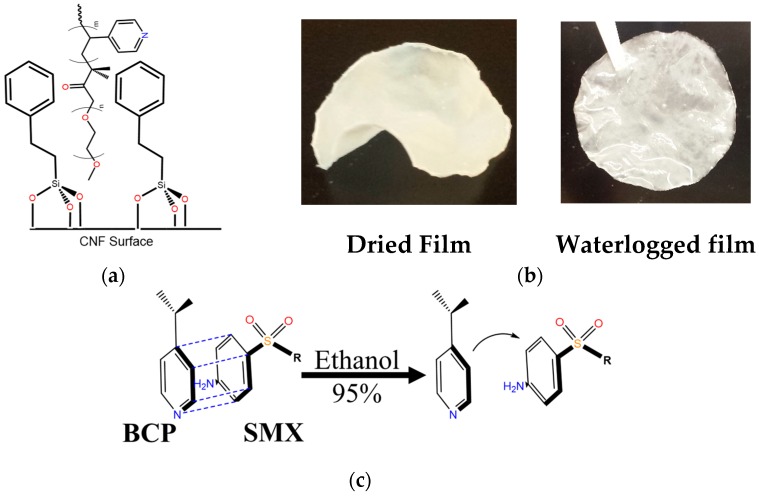
(**a**) Molecular structure of TMPES covalently attached to the surface of cellulose nanofibers (CNFs) showing the presence of P4VP-PEO; (**b**) photographs illustrating the dried and waterlogged CNFs films; (**c**) schematic of the electron-donor-acceptor (EDA) interaction of the pyridyl ring of P4VP-PEO with the substituted phenyl group of SMX and its elution with ethanol.

Once modified, the TMPES-CNFs composite can provide a support for the active adsorbent, P4VP-PEO (Figure 1b). P4VP-PEO is a block copolymer that is responsive to pH due to protonation/deprotonation of the pyridyl group. Also, slight changes in temperature can promote intermolecular interactions of the ethylene oxide chains [36]. Here, we take advantage of the high electron density of the pyridyl groups of the block copolymer to adsorb low electron density EOCs, such as SMX. By means of electron-donor-acceptor (EDA) interactions [37], it is possible to remove the SMX from aqueous solution. Since these types of interactions are reversible, it would enable the reversed adsorption reaction with a simple solvent exchange to allow the reuse of the material (Figure 2c). The addition of solid P4VP-PEO was performed during the reaction of silanols with CNFs. This mixture led to the formation of a matrix where the hydrophilic ethylene oxide chains interacted with CNFs and the vynilpiridyl functionalities interacted with the phenyl groups of the silane (Figure 2a). Figure 2b shows the actual CNFs films after the modification. It can also be noted that after immersion in water, films can adsorb the liquid despite the hydrophobic modification. Interestingly, when CNFs films without modification were shaken in water at 250 rpm for 24 h, the films dispersed in the solution, while TMPES-modified CNFs films remained undamaged (Figure S1).

### 3.2. Characterization of the Modified CNFs Films 

**Figure 3:**
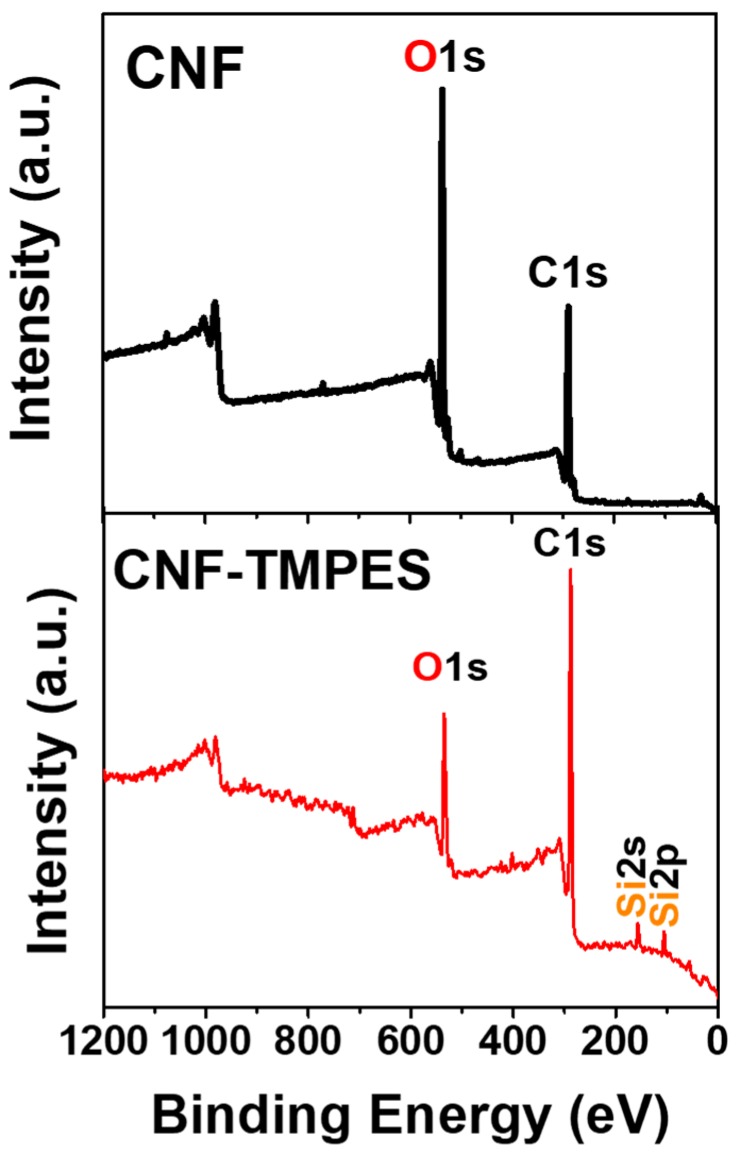
XPS spectra of CNFs and TMPES-modified CNFs films.

CNFs and TMPES-modified CNFs were characterized using XPS (Figure 3) in order to identify the silicon signal related to the Si–O–C covalent bond formed during the modification of CNF with the alkoxysilane. The XPS spectrum of the TMPES-modified CNFs clearly shows peaks corresponding to Si 2s at around 150 eV and Si 2p at 100 eV. These peaks were not present in the XPS spectra of CNFs, confirming the successful addition of TMPES.

**Figure 4:**
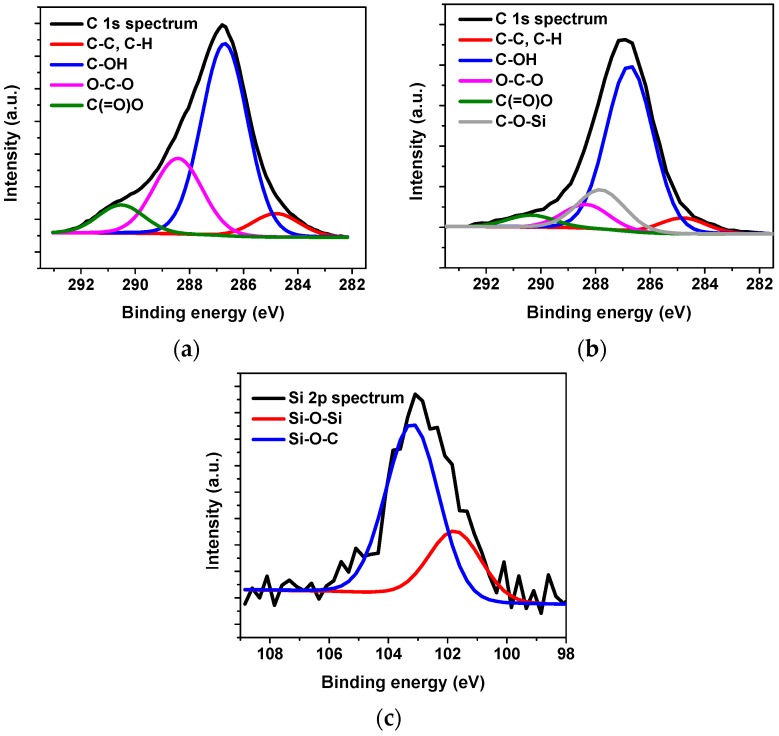
Fitted high resolution C 1s XPS spectra of CNFs (**a**) and TMPES-modified CNFs films (**b**). Fitted high resolution Si 2p XPS spectra of TMPES-modified CNFs films (**c**).

Deconvolution of the C 1s XPS spectrum of CNFs films showed four peaks that are characteristic of cellulose materials [38] (Figure 4a). The peak at 284.8 eV corresponds to C–C and C–H bonds. The peak at 286.7 eV is related to a carbon atom bonded to a single oxygen atom. For cellulose, this peak is the most intense due to the large number of hydroxyl groups in its structure. The peak at 288.4 eV corresponds to a carbon atom bonded to two oxygen atoms. The peak at 290.4 eV represents a carbon atom linked to one carbonyl oxygen and one non-carbonyl oxygen. The fitted C 1s XPS spectrum of TMPES-modified CNFs films showed the same peaks as CNFs, with the addition of one new peak at 287.8 eV (Figure 4b). This peak is possibly related to the new bond between Si–O–C obtained after modification with the alkoxysilane. This type of bond is also found in the deconvolution of Si 2p spectrum of TMPES-modified CNFs films (Figure 4c). Here, the peak at 103.2 eV represents one silicon atom bonded to one oxygen atom that at the same time is linked to one carbon (Si–O–C) [39]. In addition, the peak at 101.7 eV suggests that there is polymerization of the silane on the surface of CNFs, due to the formation of Si–O–Si bonds [40]. However, this polymerization was small compared to the formation of the covalent bonds with CNFs if we consider the intensity of the Si–O–Si and Si–O–C peaks.

**Figure 5:**
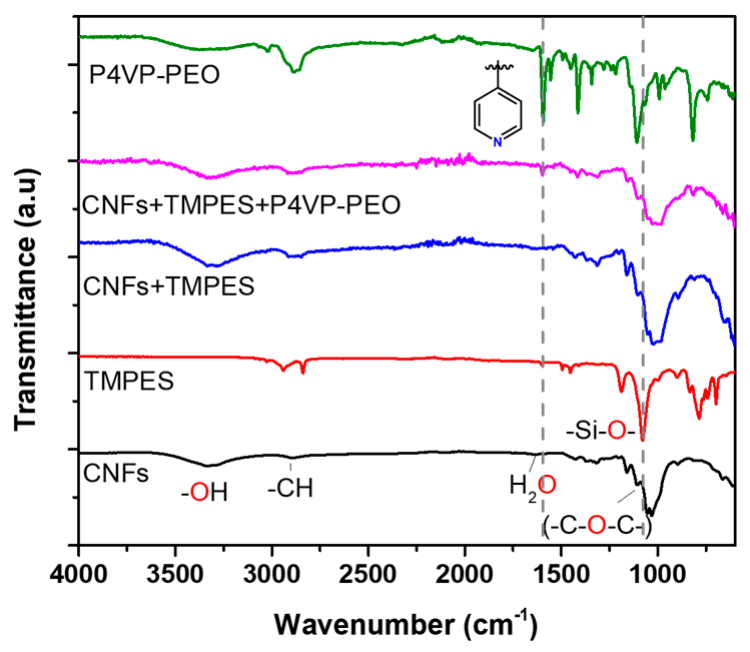
FTIR spectra of CNFs, TMPES, P4VP-PEO, CNFs + PEMS and CNFs + PEMS + P4VP-PEO.

FTIR was used to identify the critical functional group vibrations of the different components of the modified CNFs films compared to those of the precursors (CNFs, TMPES, and P4VP-PEO). Figure 5 shows the spectra of CNFs, highlighting their characteristic functional groups. A signal observed at 3300 cm^−1^ is assigned to the hydroxyl stretching vibration (–OH), the band at 2800 cm^−1^ corresponds to the stretching vibrations of the methylene groups (–CH_2_), while a sharp band around 1100 cm^−1^ corresponds to the glycosidic ring stretching. The presence of a small band around 1650 cm^−1^ which is related to adsorbed water is also common. Despite the modification of CNFs with TMPES, the FTIR spectrum of these films did not show any band related to the phenyl groups of the silane around 1450 cm^−1^. These bands were overlapped by the region of the CNFs hydrogen bending that ranges from 1300 to 1500 cm^−1^. The same effect occurred for the silicon signal expected from 500 to 1000 cm^−1^ [41]. In the FTIR spectrum of the CNFs films modified with TMPES and P4VP-PEO, it was possible to identify two bands at 1415 and 1596 cm^−1^ [42]. These bands correspond to the vibrations of the pyridine ring that were also present in the spectrum of P4VP-PEO. This is clear evidence that suggests the successful addition of P4VP-PEO to the CNFs films.

**Figure 6:**
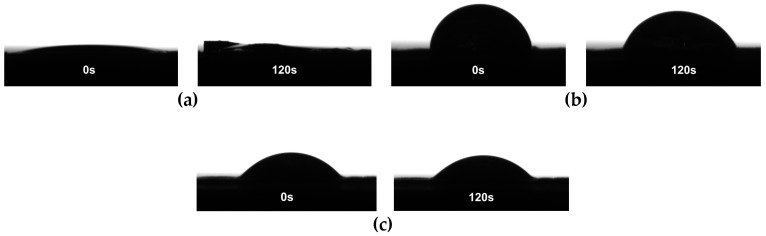
Contact angle measurements of: (**a**) CNFs films, (**b**) TMPES-modified CNFs films, and (**c**) TMPES and P4VP-PEO-modified CNFs films.

Contact angle (CA) measurements were used to estimate the wettability of CNFs films and CNFs with the respective modifications (Figure 6). The phenyl group in TMPES acts as a hydrophobic group that makes the solvation of cellulose fibers difficult. This behavior can be observed in Figure 6b, which shows a CA of 85° for TMPES-modified CNFs films at time 0. After 120 s, the value decreased to 66° due to water penetration via diffusion through the film. This result is directly related to the ratio of TMPES/CNFs used for the modification that is critical to gradually wet the films and avoid their dispersion in aqueous solution. In contrast, the water droplet penetrated the CNFs film without modification almost immediately after it came in contact with the surface. After 120 s, the film started to separate from the surface. This phenomenon is a consequence of the swelling of the material due to the high hydrophilicity of cellulose that leads to the penetration of water molecules [43]. TMPES and P4VP-PEO-modified CNFs films had higher wettability compared to CNFs films modified with TMPES alone. Here, the CA was 56°, which remained steady during the measurement. This increase in the CA was likely an effect of the polyethylene oxide chains in P4VP-PEO which add hydrophilicity to the films [44].

**Figure 7:**
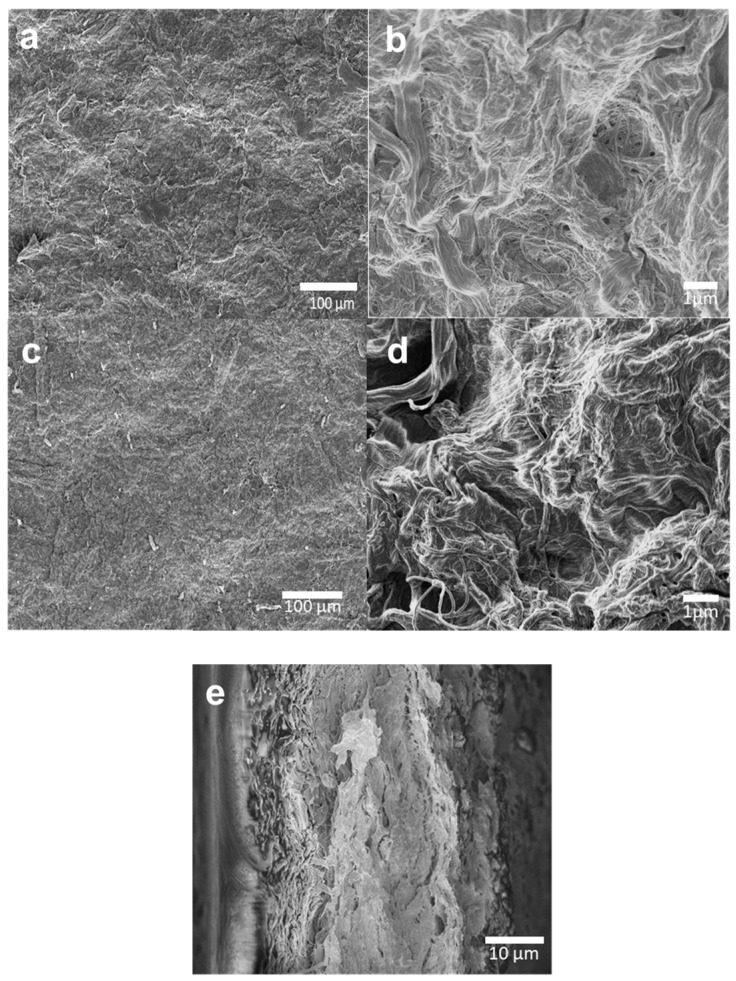
SEM micrographs of (**a**) front, (**c**) back, and (**e**) cross-section of TMPES and P4VP-PEO-modified CNFs films. Higher magnifications of the front and back are shown in (**b**) and (**d**), respectively.

SEM micrographs of TMPES and P4VP-PEO-modified CNFs films showed a rough morphology along the surface (Figure 7). Closer inspection of the images revealed that the surface was composed of tightly agglomerated fibers that were randomly distributed. This is an effect of the method used to prepare the films. Initially, components are suspended in the solution, but once the vacuum filtration begins, modified cellulose fibers accumulate layer-by-layer as shown in the cross-section image of the films (Figure 7e). These micrographs also revealed that the films are quite monolithic with minimal observable porosity, suggesting that only P4VP-PEO found in the surface of the films is readily available to interact with SMX.

### 3.3. Adsorption Batch Experiments with SMX

**Figure 8:**
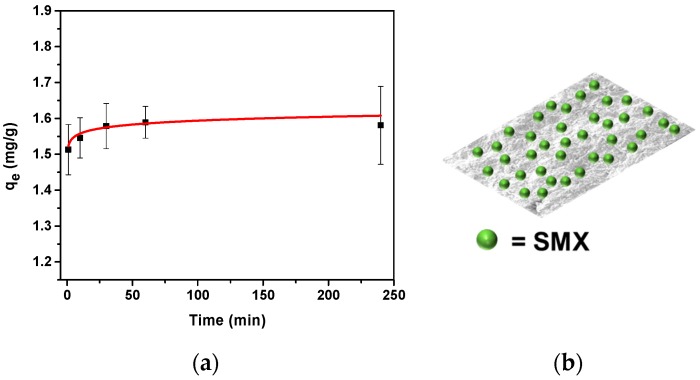
Adsorption capacity of TMPES and P4VP-PEO-modified CNFs films as a function of time (**a**), with a representation of the superficial adsorption of SMX (**b**).

Batch experiments were performed in order to assess the adsorption capacity of TMPES and P4VP-PEO-modified CNFs films against SMX as an EOC model compound. Batch experiments using TMPES-modified CNFs resulted in a negligible adsorption, which suggests that only P4VP-PEO is able to adsorb SMX (Figure S2). The effect of equilibration time on the adsorption of SMX using TMPES and P4VP-PEO-modified CNFs films is shown in Figure 8. This graph shows low *q*_e_ when the equilibrium adsorption time was set to 1 min. However, taking the measurement error into consideration, the difference between this point and higher equilibration times is minimal; therefore, most of the adsorption could be occurring on the surface of the films. This outcome is supported by the CA measurement data and SEM images that showed low water penetration and a tightly packed structure, respectively. Figure 8b shows a representation of SMX adsorbed on the surface of the modified CNFs films where P4VP-PEO is readily available. If we follow the trend line of the plot in Figure 8, the optimal adsorption equilibrium time was reached at approximately 60 min. After this point, higher equilibrium times did not cause a significant change in *q*_e_ Consequently, 60 min was used as a default adsorption equilibrium time for further batch adsorption experiments.

The adsorption isotherm of SMX using TMPES and P4VP-PEO-modified CNFs films was obtained at the equilibrium time of 1 h, neutral pH, and room temperature. After determining the *q*_e_ and the SMX equilibrium concentration (*C*_e_), the resulting plot was fitted with the Freundlich mathematical model. The Langmuir model was also utilized, but a poor fit suggested a distribution of active adsorbent sites rather than formation of a homogeneous monolayer at adsorption equilibrium [45]. The Freundlich isotherm model is an empirical equation introduced to describe the processes of adsorption. In this expression,

(2) qe=KfCe1n

**Figure 9:**
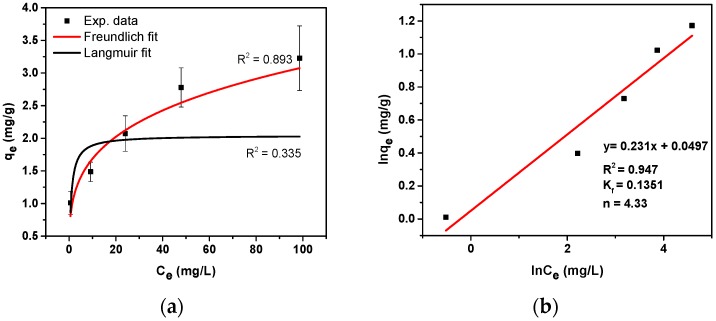
Adsorption isotherm of SMX using TMPES and P4VP-PEO-modified CNFs films fitted with the non-linear forms of the Freundlich and Langmuir models (**a**) and the linear form of the Freundlich model (**b**).

*q*_e_ represents the equilibrium adsorption amount (mg/g), *K*_f_ and *n* are empirical constants that are related to the adsorption magnitude and effectiveness, and *C*_e_ is the concentration in equilibrium (mg/L). Figure 9a shows the non-linear fitting of *q_e_* as a function of *C*_e_. This fitting suggested that the modified film had not reached its maximum adsorption capacity. Unfortunately, we were not able to determine the maximum adsorption capacity for two reasons: that is, at higher concentrations of SMX is unstable in aqueous solution (see Figure S3) and a reduction of the mass of the films used in the adsorption experiment may result in higher deviations. The linear form of the Freundlich equation,

(3) $$\ln q_{e}=\ln K_{f}-\frac{1}{n}\ln C_{e}$$

can be used to determine *K*_f_ and *n* (Figure 9b). In this case, *n* was determined to be approximately 4.3. In general, values of *n* > 1 are common and suggest that the adsorption proceeded by physical interactions, and is consistent with our hypothesis regarding EDA interactions between SMX and modified CNFs. Moreover, *n* values between 1 and 10 can generally be related to satisfactory adsorptions [46].

**Figure 10:**
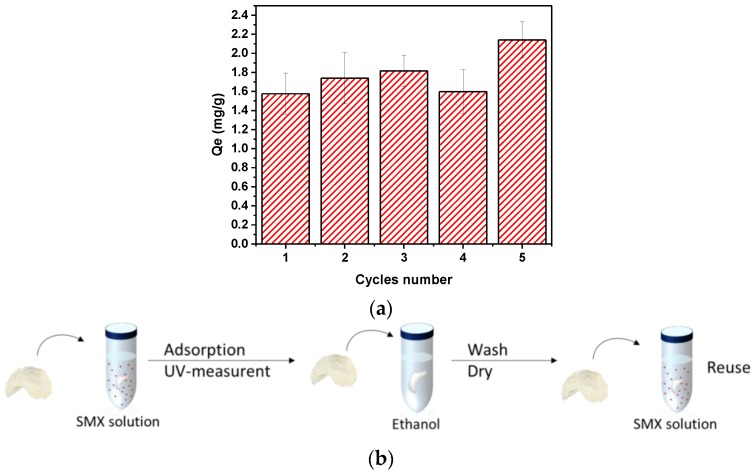
Adsorption capacity of TMPES and P4VP-PEO-modified CNFs films after different adsorption cycles of 25 ppm SMX for 1 h equilibrium time (**a**). Schematic of the reusability process of the modified films (**b**).

In terms of the reusability, Figure 10 indicates that TMPES and P4VP-PEO-modified CNFs films can be reused with negligible loss in adsorption capacity under the conditions studied. In fact, a small increase in the adsorption capacity could be appreciated after several cycles. This phenomenon may be related to the swelling of the CNFs fibers due to the high concentration of ethanol (95%) used to elude SMX [47]. Presumably, this swelling caused the P4VP-PEO molecules that were initially inaccessible to be exposed; therefore, they became available as new active sites for SMX adsorption. Moreover, a FTIR analysis of films after adsorption and elution with ethanol did not show any measurable presence of SMX (Figure S4). This result suggests that SMX is almost completely removed from the surface of the films using this method.

## 4. Conclusions

In this research, we successfully prepared a novel composite film through the modification of readily available and eco-friendly CNFs to adsorb SMX and, potentially, any pollutant with electron deficient aromatic structures. The modification of CNFs with the silane TMPES was critical to obtain a cellulose-based material that otherwise would be unstable in aqueous solution. The block copolymer P4VP-PEO dispersed on the surface of the films proved to be suitable for adsorbing SMX by means of EDA interactions. Interestingly, this type of interaction can allow a simple release of the adsorbed contaminant by elution with ethanol; consequently, it is possible to reuse the films. Characterization of the material showed that the films consisted of CNFs fibers tightly accommodated in layers. This likely translates to adsorption active sites that are mostly available on the surface of the films. In addition to this, the morphology of the films could limit the diffusion of SMX during the adsorption process. This could explain why the films reached the maximum adsorption capacity at short equilibration times. Fitting the adsorption isotherm with the Freundlich mathematical model suggested that the adsorption on the surface of the films was not homogeneous, and this could be directly related to distribution of the available P4VP-PEO. More interestingly, these films were shown to be reusable after several cycles without losing their adsorption capacity. Overall, the chemical characteristics and adsorptive behavior obtained with the films studied will contribute positively to the development of competitive materials for possible large-scale water remediation of EOCs.

## Supplementary Materials

The following are available online at http://www.mdpi.com/1996-1944/12/2/230/s1, Figure S1: (a,c) CNFs film with and without TMPES, respectively; (b,d) films after shaking in water for 24 h at 250 rpm and 25 °C, Figure S2: UV-Vis spectra of SMX, And SMX after 24 h contact time with TMPES-modified CNFs films without and with P4VP-PEO, Figure S3: (a) Freshly prepared 200 ppm SMX solution; (b) 200 ppm SMX solution after 2 weeks; (c) FTIR spectra of SMX powder from the bottle and lyophilized 2 weeks 200 ppm SMX solution; (d) SMX Structure, Figure S4: FTIR spectra of TMPES and P4VP-PEO-modified CNFs film after synthesis (unused) (a), after 3 reusability cycles in water (control) (b), and after 3 reusability cycle in 25 ppm SMX solutions (c).
