# Supplementary material for: Nanocellulose-Block Copolymer Films for the Removal of Emerging Organic Contaminants from Aqueous Solutions
Source: Materials (Basel). 2019 Jan 11;12(2):230. doi: 10.3390/ma12020230 (PMC6357086; doi:10.3390/ma12020230)
Supplement: Supplementary file 1 [file materials-12-00230-s001.pdf]

materials

MDPI

Supplementary Information

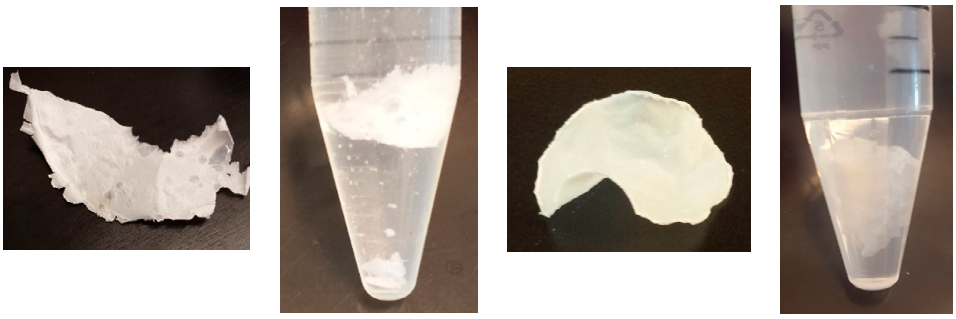

(a) (b) (c) (d)

Figure S1. (**a**,**c**) CNFs film without and with TMPES, respectively; (**b**,**d**) films after shaking in water for 24 h at 250 rpm and 25 °C.

Solutions of CNFs in ethanol/water 80/20 with and without TMPES were sonicated to obtain a homogeneous dispersed mixture. Films were prepared by vacuum filtration and placed in an oven at 110 °C for 2 h. Simple inspection of CNFs films with TMPES showed a more robust material compared to those without TMPES which were easily breakable. The stability of the CNFs films was verified by immersion in water for 24 h at 250 rpm shaking. CNFs films without TMPES started to degrade due to dispersion in the water, while CNFs films with TMPES appeared undamaged.

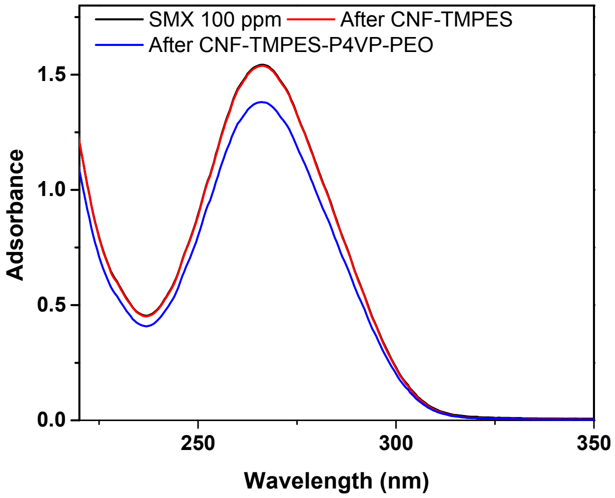

Figure S2. UV-Vis spectra of SMX, And SMX after 24 h contact time with TMPES-modified CNFs films with and without P4VP-PEO.

A Thermo Scientific Genesys 10S UV-Vis spectrometer was used to measure the absorbance of 100 ppm SMX solutions before and after they were placed in contact with CNFs films with and without TMPES for 24 h under shaking at 250 rpm and 25 °C. Absorbance in UV-Vis spectrum of SMX after 24 h contact time with TMPES and P4VP-PEO-modified CNF film was significantly lower compared to the SMX reference spectrum corresponding to a adsorption capacity of 3.2 mg of SMX/g of material. On the other hand, the absorbance of SMX solution that was in contact with TMPES-modified CNF film was similar to the reference spectrum and corresponded to 0.2 mg of SMX/g of material.

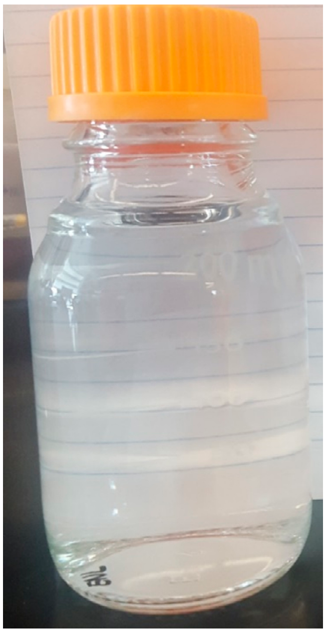

(**a**)

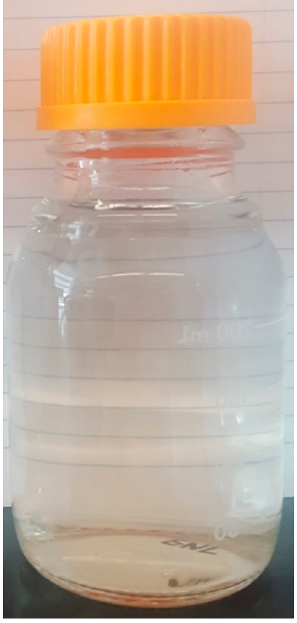

(**b**)

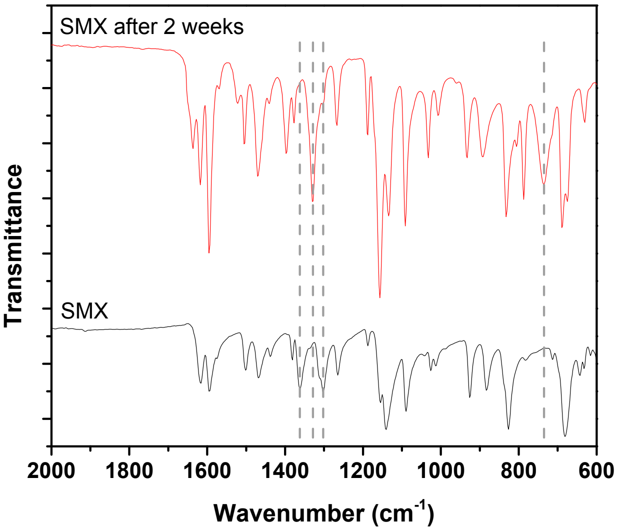

(**c**)

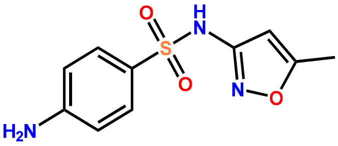

(**d**)

Figure S3. (**a**) Freshly prepared 200 ppm SMX solution; (**b**) 200 ppm SMX solution after 2 weeks; (**c**) FTIR spectra of SMX powder from the bottle and lyophilized 2 weeks 200 ppm SMX solution; (**d**) SMX Structure.

Sonication was used to dissolve SMX in water for concentrations higher than 100 ppm. These solutions appeared clear and colorless. However, after several days a yellow hue was observed. Fourier transform infrared (FTIR) spectra of SMX from the bottle and lyophilized SMX solution were recorded on a Bruker Tensor 27 using attenuated total reflectance mode (ATR) with a resolution of 4 cm^{-1}, and accumulation of 32 scans.

FTIR spectra of Figure S3 shows the spectral window from 600 to 2000 cm^{-1}. Highlighted bands can be associated with the degradation of SMX.

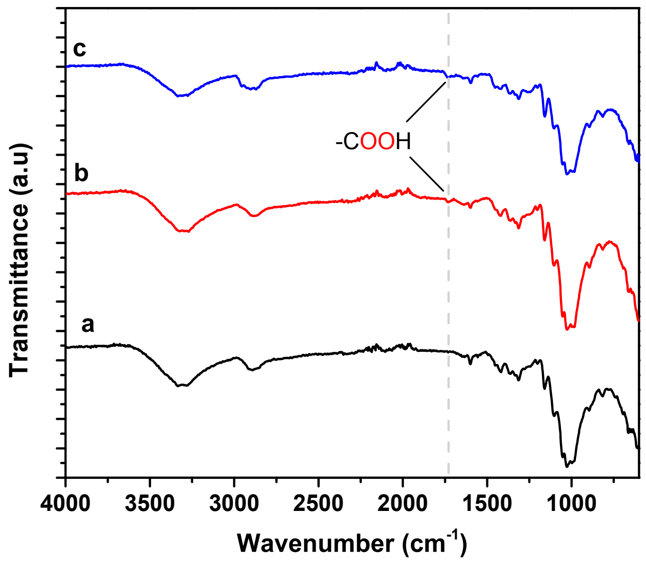

Figure S4. FTIR spectra of TMPES and P4VP-PEO-modified CNFs film after synthesis (unused) (**a**), after 3 reusability cycles in water (control) (**b**), and after 3 reusability cycle in 25 ppm SMX solutions (**c**).

The reusability of the TMPES and P4VP-PEO-modified CNFs films was tested by immersion of the samples in 95 wt.% ethanol to elude the adsorbed SMX. The same procedure was applied to control samples. The elution time in ethanol was set at 1 h with continuous shaking of 250 rpm at 25 °C. Afterwards, films were rinsed with 200 mL of water to remove the remnant ethanol, dried with compressed air until constant weight and used for another batch adsorption cycle. Fourier transform infrared (FTIR) spectra of TMPES and P4VP-PEO-modified CNFs films were recorded on a Bruker Tensor 27 using attenuated total reflectance mode (ATR) with a resolution of 4 cm^{-1}, and accumulation of 32 scans. The sample consisted of unused films, control films (three cycles in water), and films used for three adsorption cycles. A new band around 1730 cm^{-1} was observed in the spectra of the control film and the film used for the adsorption of SMX. This band cannot be assigned to any interaction of the films with SMX, since it also presented in the control. We believe that this band is the result of a slow oxidation of CNFs that implies the formation of carboxyl groups.
